# Synergistic benefits of olive pomace and multi-enzyme supplementation on fattening rabbit health and performance

**DOI:** 10.3389/fvets.2025.1710920

**Published:** 2025-12-02

**Authors:** Manal R. Bakeer, Ehab El-Haroun, Sameh A. Abdelnour

**Affiliations:** 1 Department of Physiology, Faculty of Veterinary Medicine, Cairo University, Giza, Egypt; 2 Department of Integrative Agriculture, College of Agriculture and Veterinary Medicine, United Arab Emirates University, Al Ain, United Arab Emirates; 3 Animal Production Department, Faculty of Agriculture, Cairo University, Cairo, Egypt; 4 Department of Animal Production, Faculty of Agriculture, Zagazig University, Zagazig, Egypt

**Keywords:** olive pomace, Kemzyme, multi-enzyme complex, metabolic activity, byproduct utilization, digestive enzymes, villus length

## Abstract

**Introduction:**

Disposing of agricultural and forestry waste through incineration, as is common in current waste management practices, exacerbates environmental pollution. The olive oil industry produces substantial byproducts, including olive pomace (OP). Finding more cost-effective and environmentally friendly uses for these byproducts can help reduce waste, enhance resource efficiency, and promote a circular economy. This study evaluated the physiological response of rabbits, including metabolic parameters, digestive enzyme activities, and caecal fermentation characteristics, to diets partially substituted with yellow corn, OP, and/or a multi-enzyme complex (Kemzyme, KE).

**Methods:**

A total of 120 New Zealand white rabbits were divided into four groups (30 rabbits each). The control group (CON) received a basal diet, the olive pomace group (OP) had 10% olive pomace added to their feed, while the enzyme group (KE) received 0.5% Kemzyme and the combination group (OP+KE) obtained both supplements.

**Results:**

Body weight increased significantly more in the OP+KE and KE groups compared to the other groups (*p* < 0.05). However, all supplemented groups had higher blood glucose and digestive enzyme (lipase and protease) activities compared to the control group (*p* < 0.05). Notably, total protein and DNA concentrations in duodenal tissue were augmented in the KE and OP+KE groups compared to the others (*p* < 0.05), suggesting enhanced cellular proliferation. Supplementing rabbit diets with enzymes, either alone or combined with OP, significantly increased total volatile fatty acid concentrations in the cecum compared to the control group (*p* < 0.05). All supplemented groups showed a significant improvement in butyric (*p* < 0.001) and propionic (*p* < 0.01) acid levels, coupled with a notable reduction in ammonia (*p* < 0.05). Furthermore, enzyme inclusion in the diet enhanced villus height and Brunner's gland size (*p* < 0.05) compared to the other groups.

**Discussion:**

These findings indicate that supplementing rabbit diets with Kemzyme, either alone or alongside olive pomace, effectively improves metabolic parameters, digestive enzyme activities, and intestinal morphology. This represents a sustainable advancement in rabbit nutrition, integrating agricultural byproducts with enzyme technology.

## 1 Introduction

Globally, the annual generation of vast quantities of food waste and byproducts poses considerable environmental and economic challenges. This concern has spurred initiatives within the food industry to establish “zero waste food systems,” aligning with the strategic frameworks of the European Union (EU) Circular Economy Action Plan and the European Bioeconomy Strategy (1). Virgin olive oil production, with its rapid processing, generates substantial quantities of waste materials (like olive leaves and wood) and byproducts (such as olive pomace and wastewater). These pose considerable environmental challenges, particularly in Mediterranean regions (2). Mediterranean countries account for over 90% of global olive oil production, an industry that yields vast quantities of diverse by-products. The olive milling sector alone generates more than 9 million tons of by-products annually (3). Among these byproducts, olive pomace (OP) is a semi-solid residue derived from virgin olive oil extraction processes (4), which are currently classified as environmental pollutants requiring special waste disposal. OP exhibits a heterogeneous composition comprising olive pulp and stone fragments (5), represents a valuable reservoir of diverse bioactive compounds (4). Olive pomace (OP) is rich in bioactive compounds, including various polyphenolic compounds such as hydroxytyrosol and tyrosol derivatives, iridoid precursors, secoiridoids and their derivatives, flavonoids, lignans, and phenolic acids. Additionally, OP contains significant levels of tocopherols and tocotrienols (6), along with dietary fibers, essential minerals, oligosaccharides, and beneficial monounsaturated and polyunsaturated fatty acids (7).

The utilization of olive pomace has been extensively documented in the formulation of paste and baked goods, attributable to its rich profile of polyunsaturated fatty acids, phenolic compounds, and dietary fibers, which contribute to the development of nutritionally enhanced food products and/or extend their shelf stability (8). Olive pomace (OP) has also been added to animal feed to boost the quality of animal products (3). Because of their strong antioxidant and antimicrobial properties, phenolic-rich extracts from OP have been used in vegetable oils, fish burgers, fermented milk products, and edible fruit coatings (9). This means the main goals for using olive pomace and its extracted bioactive compounds are to improve oxidative stability, increase nutritional value, and extend the shelf life of food products (4). Recent research has shown that incorporating up to 10% of olive byproduct into the diets of monogastric animals (pigs) can enhance the chemical composition and fatty acid profile of their muscles (10). Despite its benefits, OP's high fiber content could significantly limit its application as a corn replacement in monogastric animal feed.

Multienzyme complexes, typically blends of primary and supplementary enzymes or those derived from microbial fermentation, boast diverse functionalities (11). Their application in rabbit diets has been extensively investigated (3, 12), consistently showing notable improvements in nutrient digestibility and growth performance (13, 14). For instance, β-glucanase breaks down β-glucans, which are polysaccharides found in the cell walls of plants. β-glucans can increase gut viscosity, hindering nutrient absorption. β-glucanase helps reduce this viscosity, improving feed digestibility and nutrient utilization (15, 16). Cellulase hydrolyzes the β-1,4 glycosidic linkages in cellulose molecules, converting them into simpler sugars like β-glucose, or shorter polysaccharides and oligosaccharides (17). Supplementation with exogenous cellulase helps them digest fibrous plant materials, releasing trapped nutrients and improving feed efficiency in rabbits (8, 12, 14). α-Amylase breaks down starch and glycogen. It's crucial for carbohydrate digestion in animals, converting complex starches into simpler sugars that can be absorbed for energy (18). Proteases is an essential for protein digestion and absorption in animals (19). Proteases are involved in countless cellular processes, including immune response, blood clotting, and cell signaling. Industrially, they are used in detergents, food processing, and pharmaceuticals (20). Lipase hydrolyzes the ester bonds in triglycerides, releasing free fatty acids and glycerol. Critical for fat digestion and absorption in the digestive system (21), allowing the body to absorb energy from fats and utilize fat-soluble vitamins (19). However, while numerous studies have documented the benefits of multienzyme supplementation in rabbit diets for improving growth, nutrient digestibility, and overall health and well-being, their comparative effects when integrated with OP or used alone remain unexplored. The study hypothesizes that these interventions may enhance growth rates without compromising health status. Therefore, this investigation seeks to determine the effects of replacing partially yellow corn with olive pomace (OP) and/or multienzyme (Kemzyme, KE) supplementation, specifically assessing their influence on metabolic activity, body weight gain, digestive enzyme activities, and cecal fermentation characteristics in growing fattening rabbits.

## 2 Materials and methods

### 2.1 Ethical approval and materials source

The experimental protocol received approval from the Institutional Animal Care and Use Committee of Cairo University (IACUC) (Reference number: Vet CU 09092023782). Furthermore, the study was conducted and reported in accordance with the ARRIVE guidelines 2.0. Olive pomace (OP) was acquired from a two-phase olive oil extraction unit at an olive production factory in Fayoum, Egypt. The sample was immediately homogenized and stored at −20 °C until analysis. The Kemzyme was provided by Kemin Agrifoods Europe (Belgium – Herentals – EMENA Headquarters, Animal Nutrition & Health).

### 2.2 Experimental design

This study involved 120 male New Zealand White rabbits (*Oryctolagus cuniculus*), each approximately 2 months old and weighing around 1000 g ± 5.0. We acquired the experimental animals from the Faculty of Veterinary Medicine, Cairo University, and randomly assigned them to one of four groups (*n* = 30 rabbit does/group). Each group had 15 replicates, totaling 30 animals (15 replicates × 2 animals). Following a 7-day adaptation phase, all rabbits were maintained under optimal hygienic conditions and standardized laboratory parameters, receiving a basal control diet with dietary supplementation (Table 1). The experimental groups were set up as follows: (1) the control group (C) received the standard basal diet; (2) the olive pomace group (OP) was given a diet supplemented with 10% olive pomace (22); (3) the enzyme group (KE) received the basal diet supplemented with 0.5% “Kemzyme” (Kemin Agrifoods Europe, Belgium – Herentals – EMENA Headquarters, Animal Nutrition & Health) a multi-enzyme complex with beta-glucanase, cellulase, alpha-amylase, protease, and lipase; and (4) the combination group (OP+KE) received both olive pomace and Kemzyme in their diet. The experiment ran for 8 weeks (4 months of age), with fresh, potable water available at all times. Body weights were recorded at the start and conclusion of the period.

**Table 1:** Ingredients and chemical analysis of the control and experimental diets.

**Ingredients (%)**	**Control**	**OP**	**KE**	**OP+KE**
Berseem hay	30.00	29.10	30.00	29.10
Olive pomace	–	10.00	–	10.00
Barley grain	21.00	19.00	21.00	19.00
Yellow corn	5.00	3.00	5.00	3.00
Wheat bran	21.10	20.00	20.6	19.5
Kemzyme^1^	–	–	0.5	0.5
Soybean meal	17.50	13.50	17.50	13.50
Molasses	3.00	3.00	3.00	3.00
CaCl_2_	1.50	1.50	1.50	1.50
NaCl	0.40	0.40	0.40	0.40
Vitamins and Minerals Premix^†^	0.30	0.30	0.30	0.30
DL-Methionine	0.20	0.20	0.20	0.20
**Chemical analysis (%)** ^‡^				
Moisture	9.40	9.50	9.46	9.53
Dry matter	90.60	90.50	90.61	90.46
Crude protein	17.50	17.48	17.51	17.48
Crude fiber	16.00	16.30	16.39	16.32
Ether extract	2.53	2.42	2.44	2.44
Total Ash	7.10	7.15	7.23	7.12
Nitrogen-free extract (NFE)	47.47	47.15	47.16	47.13
Digestible energy, kcal/kg	2698.89	2699.60	2712.43	2701.22

†The rabbit's vitamin and mineral premix/kg contained the following vitamins and minerals: A-4,000,000 IU, D3-5000,000 IU, E-16,7 g, K-0.67 g, B1-0.67 g, B2-2 g, B6-0.67 g, B12-0.004 g, B5-16.7 g, Pantothenic acid-6.67 g, Biotin-0.07 g, Folic acid-1.67 g, Choline chloride-400 g, Zn-23.3 g, Mn-10 g, Fe-25 g, Cu-1.67 g, I-0.25 g, Se-0.033 g, and Mg-133.4 g (Rabbit premix). ^‡^Official methods of analysis of AOAC (24). ^1^Kemzyme (Kemin Agrifoods Europe, Belgium—Herentals—EMENA Headquarters, Animal Nutrition & Health).

### 2.2 Animal housing and management

Rabbits were housed in enclosures featuring J-feeders and automatic watering systems (50 × 50 × 40 cm^3^ galvanized wire battery cages). The facility maintained adequate ventilation using electric fans and windows, supplemented by both natural light and fluorescent fixtures, ensuring a 14:10 h light/dark photoperiod. Ambient temperature was kept at approximately 25 °C and relative humidity at 75%. Both control and experimental diets were formulated to meet the nutritional requirements for rabbits as established by the (23). All experimental animals had unrestricted access to their respective diets. These diets underwent comprehensive chemical analysis following standardized procedures outlined by the Association of Official Analytical Chemists (24). The specific composition and chemical analysis of the experimental diets used in this investigation are detailed in Table 1.

### 2.3 . Biochemical analysis and digestive enzyme assessment

At the end of the experiment, blood specimens (*n* = 10 from each group) for biochemical evaluation were obtained from the ear vein of each rabbit following a standardized venipuncture protocol (25). Each animal was fasted for 12 h prior to this single blood collection. Samples were immediately transferred to EDTA-free vacutainer tubes (BD Vacutainer^®^, Becton Dickinson, Franklin Lakes, NJ, USA) and processed within 30 min of collection. The specimens underwent centrifugation at 2,795 × g for 10 min at 4 °C using a refrigerated centrifuge (Model 5424R, Eppendorf, Hamburg, Germany) to separate serum from cellular components. The resulting serum was aliquoted into cryovials (Cryopure^®^, Sarstedt AG & Co., Nümbrecht, Germany) and preserved at −20 °C until biochemical analysis. Following blood collection, rabbits were humanely sacrificed following the standardized protocol established by Nakyinsige et al. (26) for histological study. Serum samples were subsequently analyzed for digestive enzyme activity (lipase, amylase, and protease) using commercial enzyme-linked immunosorbent assay kits (Rabbit Digestive Enzyme ELISA Kit, MyBioSource, Inc., San Diego, CA, USA) according to the manufacturer's specifications. Additional biochemical parameters, including total protein concentration, glucose, triglycerides, and cholesterol levels, were quantified using an automated biochemical analyzer (Cobas c311, Roche Diagnostics GmbH, Mannheim, Germany) with appropriate quality controls and calibration standards (PreciControl ClinChem Multi 1 and 2, Roche Diagnostics GmbH, Mannheim, Germany).

### 2.4 DNA and protein analysis

Six rabbits from each treatment group were selected, subjected to a 12-hour fast, weighed, and then slaughtered according to Islamic guidelines for animal slaughtering. The slaughtering was performed by cutting the major blood vessels in rabbits. Rabbits were immediately dissected and eviscerated to collect duodenal segments. Selected duodenal specimens were then cryopreserved in liquid nitrogen (−196 C) for later analysis of DNA and protein concentrations in tissue homogenates. Other specimens were fixed in 10% neutral buffered formalin (10% NBF) for histomorphological examination at room temperature.

### 2.5 Volatile fatty acid and ammonia nitrogen analysis

The cecal contents were immediately filtered using two layers of sterile gauze. The filtered cecal fluid was then used for pH determination with a calibrated electronic digital pH meter. The filtered contents were then centrifuged at 7,000 x g for 10 min. The resulting supernatant was divided into two aliquots: one was treated with 0.2 M hydrochloric acid for ammonia nitrogen (NH3-N) quantification, while the other was preserved with 5% orthophosphoric acid (v/v) containing 1% mercuric chloride (w/v) for total volatile fatty acid (VFA) and individual VFA proportion analysis. We determined NH3-N concentrations in cecal digesta spectrophotometrically, as described by Chaney et al. (27). Total VFA concentrations were quantified via steam distillation following the protocol established by Eadie et al. (28). We used High-Performance Liquid Chromatography (HPLC; Model Water 600; UV detector, Millipore Corp.) to determine the proportional composition of individual VFAs, as outlined by Mathew et al. (29).

### 2.6 Histomorphometry analysis

Standard histological protocols were used to process the duodenal specimens (30). This involved sequential steps of rinsing with tap water, fixation in 10% neutral buffered formalin, xylene clearance, paraffin embedding, and sectioning at 3–5 μm thickness. Sections were then deparaffinized and stained with hematoxylin and eosin (H&E). Microscopic evaluation was performed using a LEICA DM500 light microscope. Digital micrographs were captured with an attached LEICA ICC50 HD camera, and morphometric analyses were conducted using Leica Microsystems' specialized image analysis software (LAS version 3.8.0) at the Veterinary Medicine College, Cairo University, Egypt.

### 2.7 Statistical analysis

Before performing statistical analysis, we checked the data for normality using the Shapiro-Wilk test and for homogeneity of variances using Levene's test. Data were subjected to one-way analysis of variance (ANOVA) using IBM SPSS Software (version 25). Tukey's post-hoc test was used for multiple comparisons between treatment means, with significance defined as *p* ≤ 0.05. Results are reported as means ± SEM.

## 3 Results

### 3.1 Metabolic parameters and body weight

**Figure 1:**
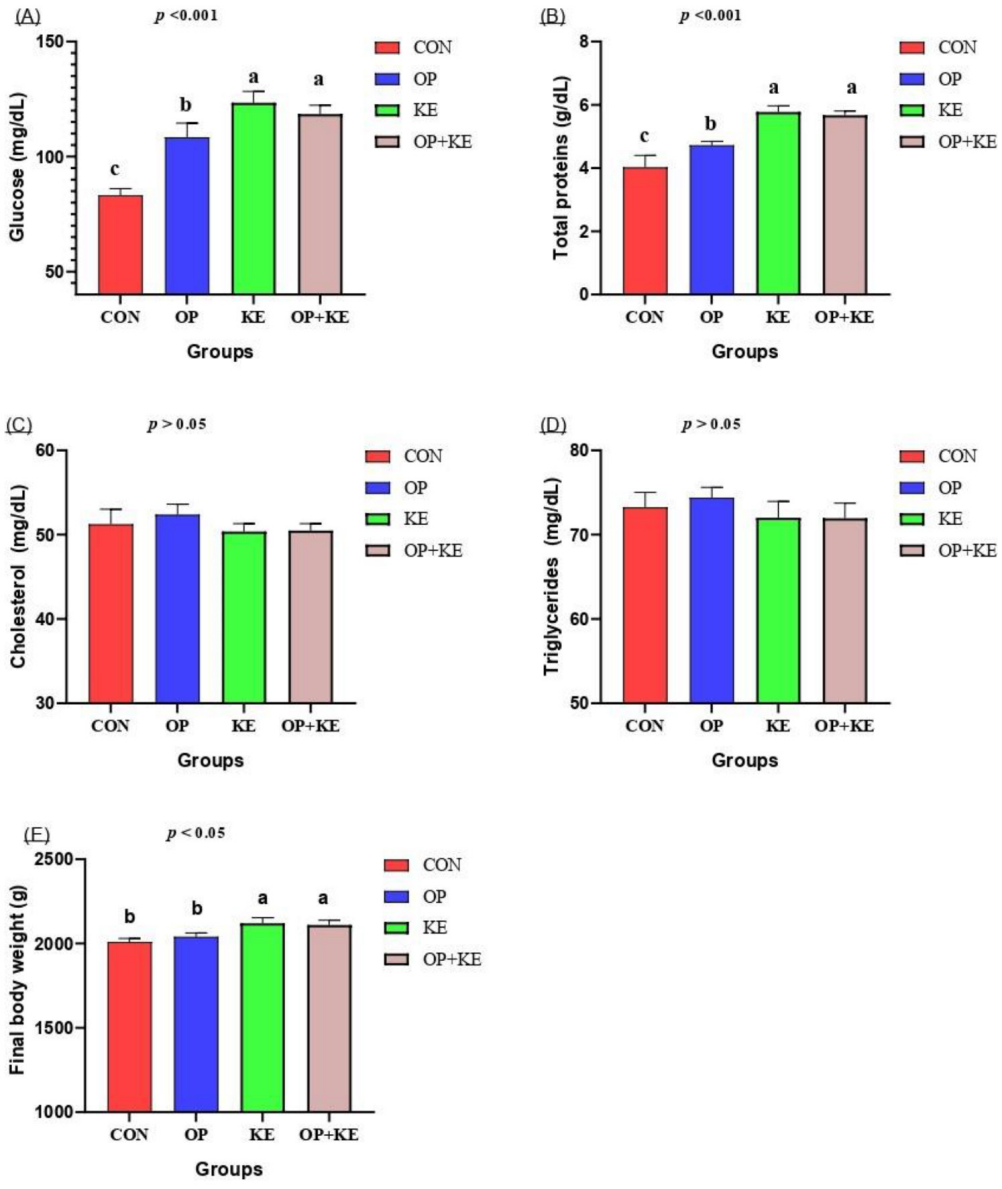
Graph showing the effect of different treatment on glucose **(A)**, total protein **(B)**, cholesterol **(C)**, triglycerides **(D)** concentration, and final body weight **(E)**. Treated groups presented as control group (CON), olive pomace (OP) supplemented group, Kemzyme treated group (KE) and the group treated with both olive pomace and kemzyme (OP+KE). Statistical significance was set at *p* < 0.05. Different letters (^a, b, c^) denote significant differences between groups. Data are shown as mean ± SE.

This research comprehensively evaluated metabolic parameters and body weight across all experimental groups (Figure 1). Serum glucose concentrations were significantly higher in rabbits supplemented with 10% olive pomace (OP), 0.5% Kemzyme (KE), and the combined olive pomace-Kemzyme (OP+KE) group compared to the control (Figure 1A) (*p* < 0.05). Total protein concentrations also showed substantial increases in both KE and OP+KE groups relative to control values (Figure 1B) (*p* < 0.05). Conversely, this study found no statistically significant differences among the experimental groups for serum cholesterol (Figure 1C), and triglyceride (Figure 1D) concentrations (*p* >0.05). Final body weights were significantly higher in both the KE and OP+KE groups compared to the other groups (*p* < 0.05), as shown in Figure 1E. Adding multienzymes alone or in combination with PO could enhance body weight by modulating serum metabolites.

### 3.2 Digestive enzymes activity

**Figure 2:**
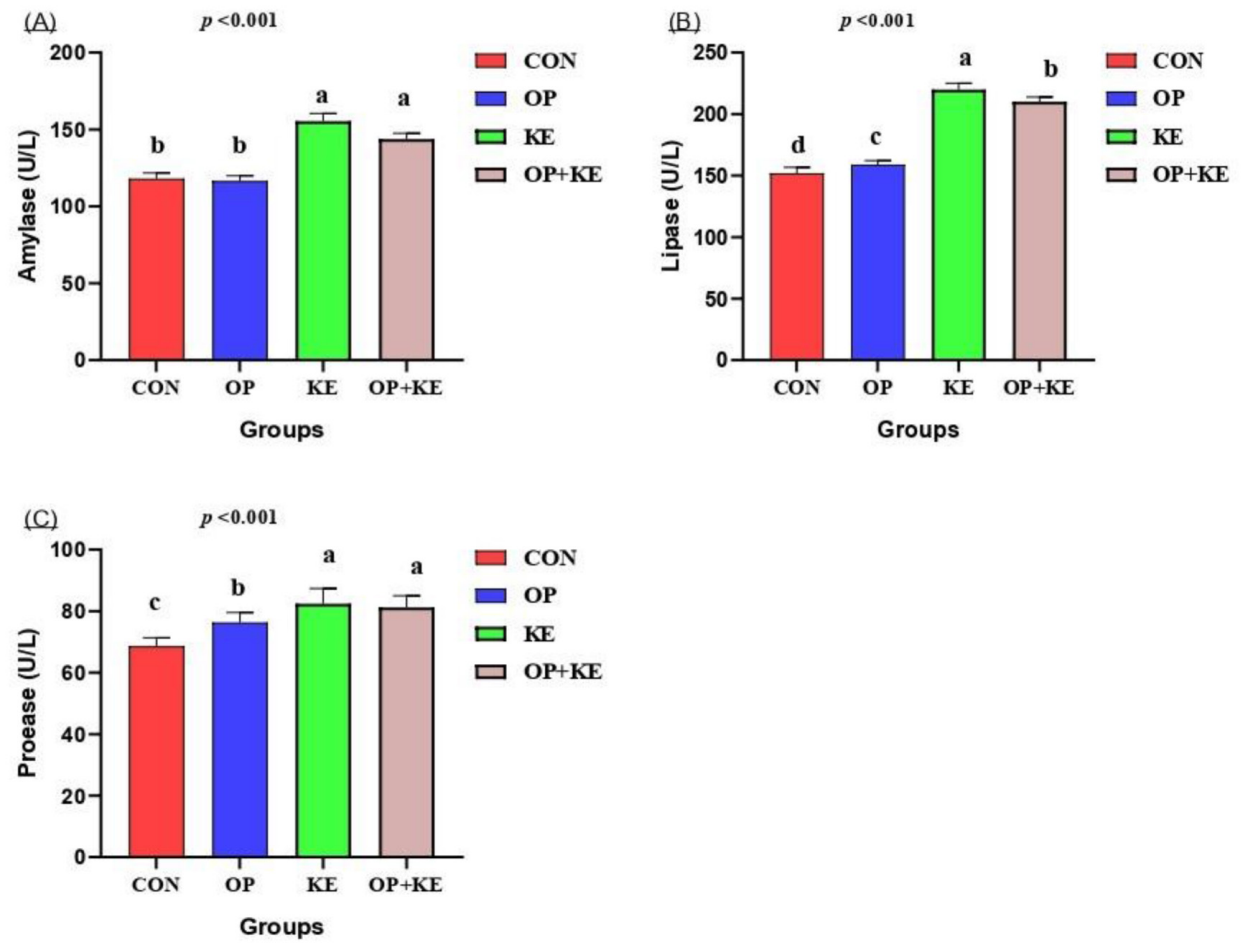
Graph showing the effect of different treatment on different digestive enzymes activity, including amylase **(A)**, lipase **(B)** and protease **(C)**. Treated groups presented as control group (C), olive pomace supplemented group (O), Kemzyme treated group (K) and the group treated with both olive pomace and kemzyme (OK). Statistical significance was set at *p* < 0.05. Different letters (^a, b, c^) denote significant differences between groups. Data are shown as mean ± SE.

The activity of amylase enzymes was significantly higher in the KE and OP+KE groups compared to the other groups (*p* < 0.05, Figure 2A). The rabbits in the KE group exhibited significantly higher lipase activity compared to the other groups (*p* < 0.05, Figure 2B). Additionally, all treated groups showed significantly elevated levels of lipase compared to the control diet (*p* < 0.05). The levels of protease were significantly greater in rabbits from the KE and OP+KE groups compared to the other groups (*p* < 0.05, Figure 2C).

### 3.3 DNA and protein quantification

**Figure 3:**
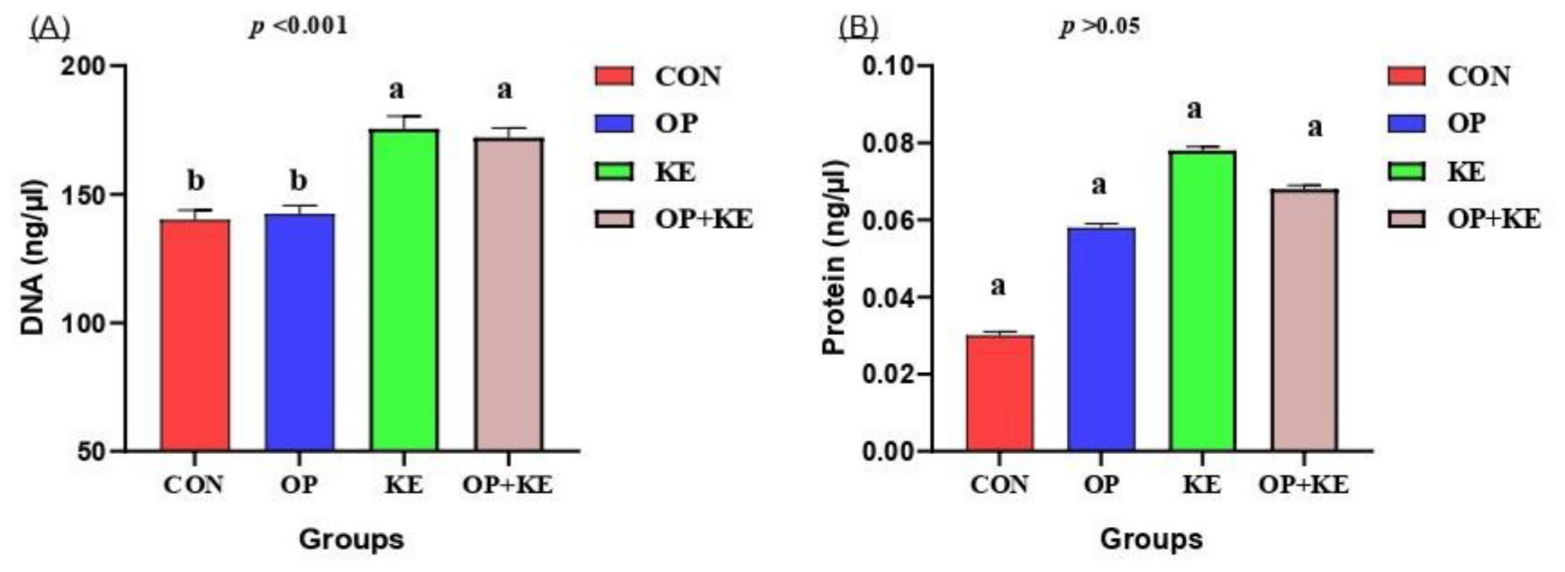
Graph showing the effect of different treatment on DNA **(A)** and protein **(B)**. Treated groups presented as control group (C), olive pomace supplemented group (O), Kemzyme treated group (K) and the group treated with both olive pomace and kemzyme (OK). Statistical significance was set at *p* < 0.05. Different letters (^a, b, c^) denote significant differences between groups. Data are shown as mean ± SE.

Analysis of duodenal tissue homogenates revealed a significant increase in DNA concentration (Figure 3A) in both the KE and OP+KE groups compared to control values, indicating enhanced cellular proliferation (*p* < 0.05). Conversely, no statistically significant differences were observed in protein concentrations (Figure 3B) among any of the experimental groups (*p* > 0.05).

### 3.4 Cecal fermentation parameters

**Figure 4:**
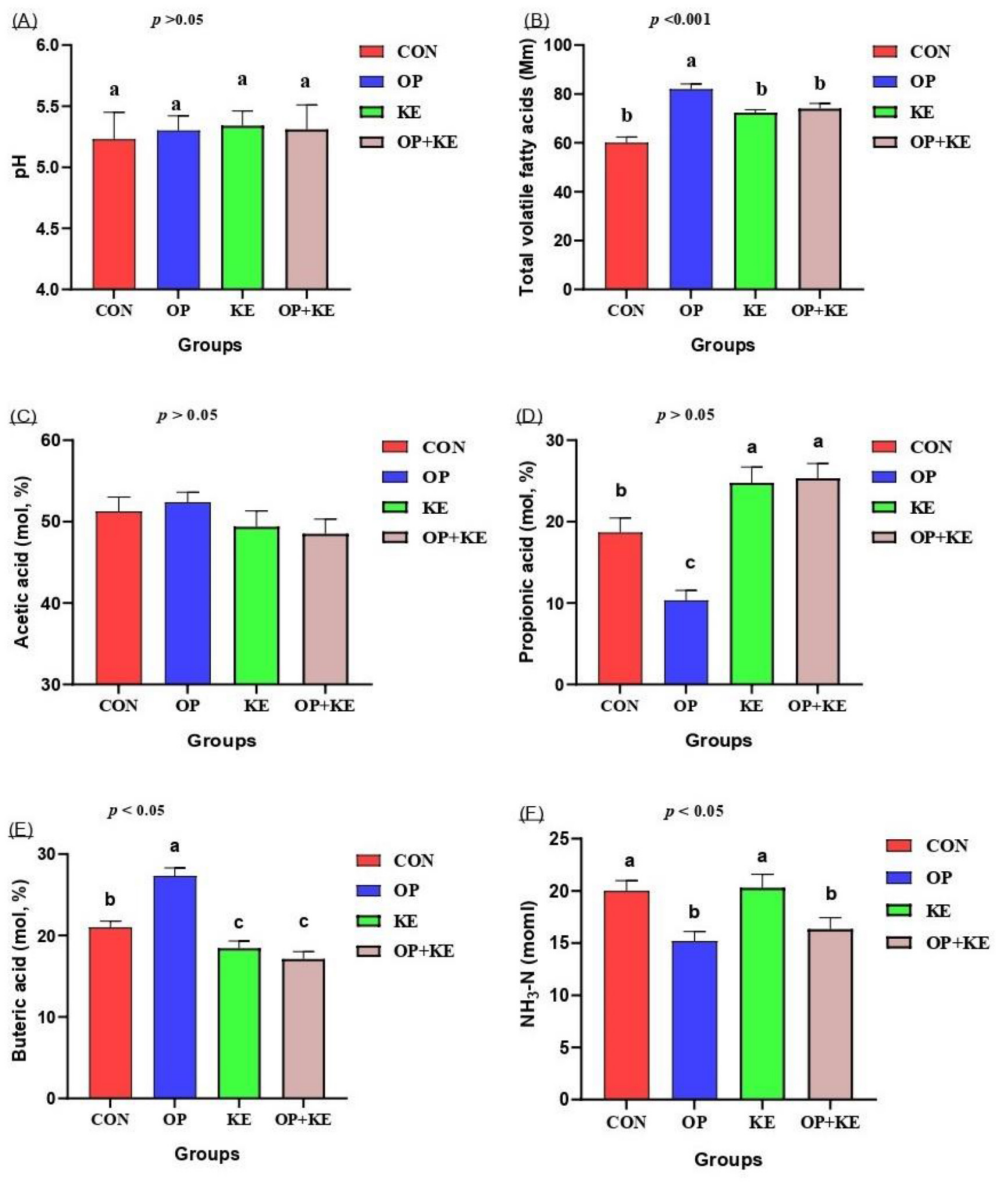
Graph showing the effect of different treatment on pH **(A)**, total VFAs **(B)**, acetic acid **(C)**, propionic acid **(D)**, butyric acid **(E)** and NH_3_-N **(F)**. Treated groups presented as control group (C), olive pomace supplemented group (O), Kemzyme treated group (K) and the group treated with both olive pomace and kemzyme (OK). Statistical significance was set at *p* < 0.05. Different letters (^a, b, c^) denote significant differences between groups. Data are shown as mean ± SE.

Cecal pH values showed no significant differences across the control and experimental groups (Figure 4A) (*p* > 0.05). However, total volatile fatty acid (VFA) concentrations were significantly higher (*p* < 0.05) in both the KE and OP+KE groups compared to the control (Figure 4B). When analyzing individual VFA proportions, acetic acid concentrations remained unchanged across all groups (Figure 4C). Propionic acid concentrations moderately increased in the KE and OP groups (*p* < 0.05) but significantly decreased in the OP group relative to the control (Figure 4D). Conversely, butyric acid concentrations were significantly (*p* < 0.05) elevated in the OP group compared to the control (Figure 4E). Finally, ammonia nitrogen (NH3-N) concentrations showed only minor, non-significant variations among the experimental groups (Figure 4F).

### 3.5 Histomorphometry analysis

**Figure 5:**
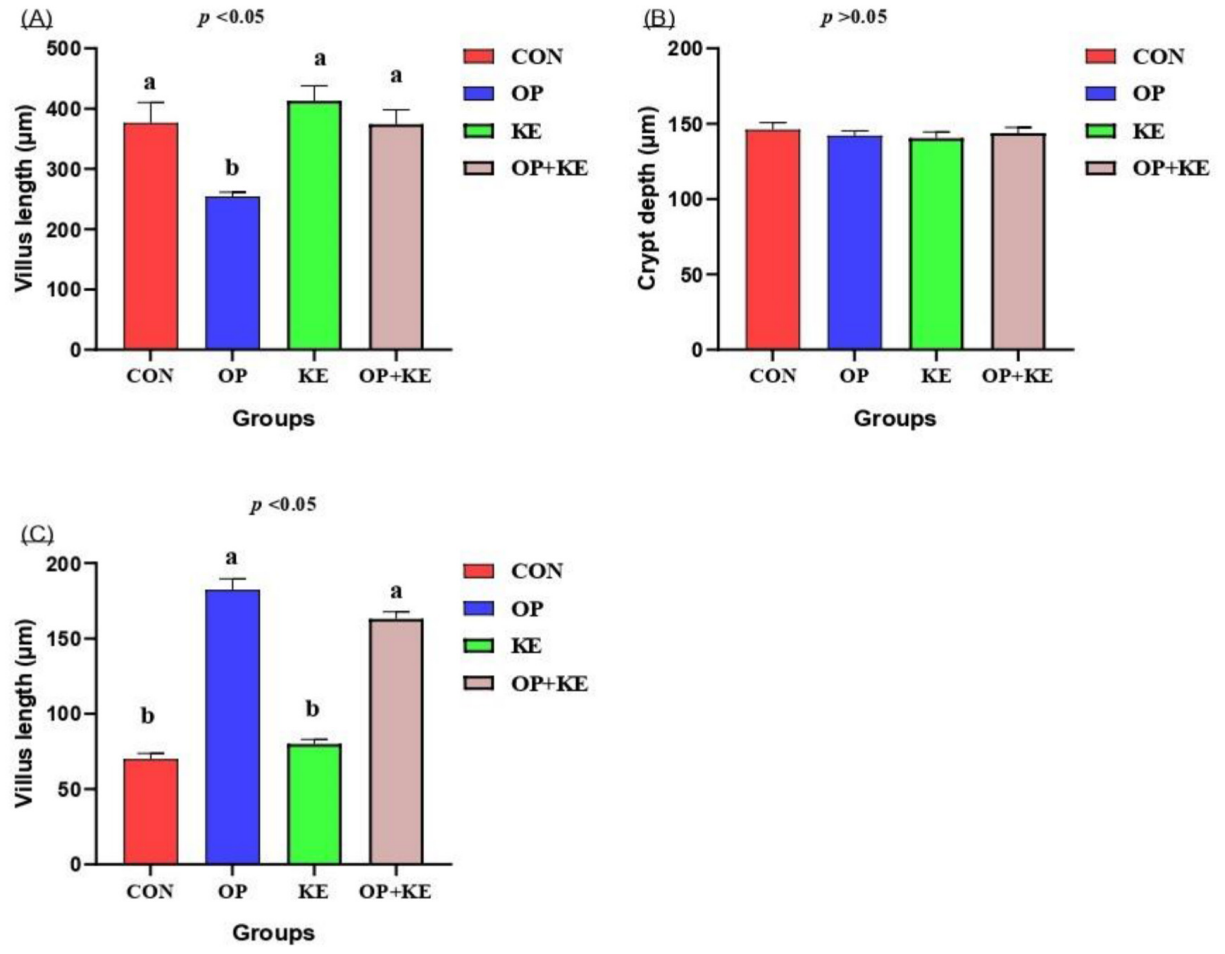
Graph showing the effect of different treatment on villus length **(A)**, crypt depth **(B)** and glands area **(C)**. Treated groups presented as control group (C), olive pomace supplemented group (O), Kemzyme treated group (K) and the group treated with both olive pomace and kemzyme (OK). Statistical significance was set at *p* < 0.05. Different letters (^a, b, c^) denote significant differences between groups. Data are shown as mean ± SE (*n* = 4).

Morphometric analysis demonstrated significant increases in villus height in both KE and OP+KE groups compared to control values (Figure 5A). However, crypt depth measurements revealed no significant differences among the experimental groups (Figure 5B). Furthermore, quantitative assessment of Brunner's gland area demonstrated significant enhancement in both KE and OP+KE groups relative to control values (Figure 5C), suggesting augmented glandular secretory capacity.

## 4 Discussion

This investigation aimed to evaluate the potential of supplementing New Zealand White rabbits with olive pomace (OP) and/or a multi-enzyme complex (Kemzyme) as viable yellow corn alternative. The study assessed their ameliorative effects on metabolic parameters, digestive enzyme activities, and intestinal morphology. These findings demonstrate significant alterations in various physiological and histological parameters that warrant comprehensive interpretation within the context of existing literature. Food industries generate substantial quantities of olive pomace, which has been successfully incorporated into animal nutrition strategies (31). Olive pomace constitutes not only a complementary energy source but also represents a significant reservoir of bioactive compounds, particularly polyphenols with potent antioxidant capacity (32). The integration of exogenous enzymes offers an effective approach to enhance rabbit digestion and improve the nutritional quality of olive pomace (31). As demonstrated in our investigation, supplementation with Kemzyme alone (KE) or in combination with olive pomace (OP+KE group) significantly improved growth performance, glucose metabolism, protein synthesis, caecal fermentation parameters, DNA and protein concentrations, and overall health status in growing rabbits. The observed elevation in serum glucose concentrations across all supplemented groups (OP, KE, and KE+OP) compared to control values suggests enhanced carbohydrate metabolism, potentially attributable to improved nutrient digestibility. These findings corroborate earlier observations reported by Attia et al. (33), who documented similar metabolic enhancements following enzyme supplementation. The multi-enzyme complex utilized in our study contains α-amylase, which facilitates starch hydrolysis and subsequent glucose absorption, potentially explaining the elevated serum glucose levels (11, 16). Based on literature (Table 2), the phenolic compounds, acids, and minerals found in olive pomace and its extract are the source of its significant biological activities. These activities, which include antioxidant and antimicrobial effects, are crucial for supporting the growth and overall health of growing rabbits.

**Table 2:** The main compounds, phenolic compounds, and acids, minerals, and identified in olive pomace and its extract based on the literature.

**Olive pomace**					
**Main compounds (g/100g)**					**Ref**.
Total lipids		2.33 ± 0.06			(62)
		12.06 ± 0.79			(63)
Moisture content		64.58 ± 2.51			(62)
Protein		2.48 ± 0.04			
		5.66 ± 0.31			(63)
Ash		1.13 ± 0.02			(62)
		4.55 ± 0.22			(63)
Total carbohydrates		29.48 ± 2.4			(62)
		20.37 ± 1.65			
**Mineral (mg/Kg)**					
Calcium		352.03 ± 23.01			(63)
Zinc		2.92 ± 0.36			
Magnesium		145.41 ± 16.54			
Iron		12.24 ± 2.71			
		1.56 ± 0.22			
**Biological activity for olive pomace extract**					
Total phenolic content		12.08 ± 1.52			(63)
Antioxidant Activity (DPPH ^*^ (%, μmol TE/g))		3.23 ± 0.10			
		1.0 ± 0.2 g TE/100 g DW			(64)
FRAP (μmol Fe(II)/g)		80.41 ± 1.12			(63)
		2.3 ± 0.1			(64)
Phenolic Compound					**Ref**.
Hydroxytyrosol		28.71 ± 0.42			(63)
		215 ± 9			(64)
Tyrosol		10.63 ± 0.06			
**Phenolic acids**		**Ref**.	**Flavonoids**		
Cinnamic acid	18.45 ± 0.95	(63)	Naringenin	125.11 ± 0.56	(64)
*p*-Coumaric acid	44.99 ± 3.01		Quercetin	168.62 ± 15.47	
Ferulic acid	22.31 ± 1.38		Hesperidin	137.30 ± 5.22	
Vanillic acid	39.51 ± 0.48				
Homovanillic acid	103.45 ± 6.24				
Rosmarinic acid	22.05 ± 0.80				
Ellagic acid	142.93 ± 4.49				
Total phenolics	2.9 g GAE/100 g DW	(64)			

Additionally, the bioactive compounds in olive pomace, particularly phenolic compounds, may modulate glucose metabolism through enhanced insulin sensitivity mechanisms, as previously reported by Lammi et al. 34], who demonstrated that olive polyphenols positively influence glucose homeostasis through multiple molecular pathways. The significant augmentation in total protein concentrations observed in the KE, and KE+OP groups aligns with findings reported by Abdel-Moneim et al. (34), who documented enhanced protein synthesis following enzyme supplementation in broilers. This effect might be attributed to the proteolytic activity of the multi-enzyme complex, which enhances protein digestion and amino acid availability for tissue synthesis. Indeed, (35) reported that exogenous enzyme supplementation significantly improves protein digestibility and nitrogen retention in monogastric animals. Furthermore, the phenolic compounds present in olive pomace have been shown to exert anti-inflammatory effects, potentially creating a more favorable intestinal environment for nutrient absorption and protein synthesis. The enhanced metabolic parameters in enzyme-supplemented groups can be attributed to the natural limitations of the monogastric gastrointestinal tract in producing sufficient endogenous enzymes for optimal digestion. As noted by Oloruntola et al. (13), dietary enzyme supplementation in monogastric animals enhances the breakdown of compounds that may not be effectively hydrolyzed by endogenous digestive enzymes, thereby improving the digestibility and absorbability of various dietary components, including carbohydrates, lipids, and proteins, ultimately enhancing animal productivity. Interestingly, neither olive pomace nor Kemzyme supplementation significantly affected serum cholesterol or triglyceride concentrations in our study. These findings contrast with previous reports by Paiva-Martins et al. (36), who documented hypocholesterolemic effects of olive byproducts in rats. This discrepancy might be attributed to species-specific differences in lipid metabolism or the relatively short duration of our experimental protocol. Additionally, the dose of olive pomace utilized in our study (10%) might be insufficient to elicit significant alterations in lipid profiles, as previous studies employed higher concentrations or longer durations (3, 37).

The moderate yet significant elevations in lipase and protease activities in KE and OP+KE groups support (38), findings on enhanced digestive enzyme activity with exogenous enzymes in poultry. Our data showed considerably higher serum protease and lipase activities in these groups, consistent with (39), who suggested exogenous enzymes persist and act in the colon. This boost in activity might also stem from an exogenous enzyme-induced favorable GI tract pH shift (40). Furthermore, the synergistic action of exogenous and endogenous enzymes likely enhanced intestinal enzymatic activities (33) either by directly increasing the enzyme pool or indirectly stimulating endogenous secretion via microbial modulation (41). Interestingly, amylase activity remained unchanged, despite the α-amylase component. This could be due to complex pancreatic enzyme regulation, where feedback inhibition occurs with high substrate availability (42).

The significant increase in DNA concentrations in duodenal tissue homogenates from KE, and KE+OP groups indicates enhanced cellular proliferation, consistent with findings reported by Mahmood et al. (43), who demonstrated that enzyme supplementation promotes intestinal epithelial cell turnover. As established by Venkatasubramanian et al. (44), the protein-to-DNA ratio serves as an indicator of cell size, while DNA concentration in tissue reflects the rate of mitosis within a cell population. The observed increases in total DNA and protein concentrations in the KE and OP+KE groups may be attributed to the presence of polyphenols, which are synthesized by plants and abundantly present in olive pomace (37, 45). Additionally, olive pomace enhances oxidation resistance, contributes to the nutritional value of food, and extends shelf-life (7, 46). These polyphenolic compounds play a significant role as suppressors of free radicals, functioning as antioxidants and modulating the synthesis of reactive oxygen species (ROS) in tissues, which might otherwise stimulate oxidative damage and DNA degradation (47). Moreover, the increased enzymatic activities observed in the intestine of Kemzyme-supplemented groups (33), which contributed to improved digestive function and body weight in the KE, and KE+OP groups, may be mechanistically linked to the elevated DNA and protein concentrations.

This effect likely contributes to improved intestinal function through enhanced absorptive capacity and barrier integrity. The lack of significant differences in protein concentrations among experimental groups, despite elevated DNA content, suggests that cellular hyperplasia rather than hypertrophy is the predominant response to the dietary interventions (48). This interpretation is further supported by our histomorphometry findings, which demonstrated significant increases in villus height without corresponding alterations in crypt depth. The caecal fermentation parameters provide valuable insights into the microbial activity within the hindgut, which significantly influences nutrient utilization and gastrointestinal health in rabbits (49). According to our current findings, total VFA concentrations were significantly higher in the KE and OP+KE groups compared to the control group, suggesting enhanced microbial fermentation activity, potentially resulting from improved substrate availability for cecal microbiota (50).

The addition of olive pomace resulted in an increase in total volatile fatty acids (TVFAs) and a decrease in NH_3_ levels, attributable to the phenolic compounds in olive derivatives that exert an antibiotic effect against intestinal or cecal pathogenic bacteria (51). Furthermore, phenolic compounds can enhance TVFA synthesis by stimulating the activity of digestive enzymes (52). The reduction in ammonia levels observed in our study likely reflects improved intestinal and cecal health, as elevated ammonia levels are often associated with microbial dysbiosis and compromised intestinal integrity. Exogenous enzymes, particularly carbohydrase, may facilitate the release of fermentable oligosaccharides from dietary fiber, thereby stimulating VFA production by hindgut microorganisms (53). The differential effects on individual VFA proportions, specifically the increased propionic acid in KE and OP+KE groups versus elevated butyric acid in the OP group, highlight substrate-specific effects on microbial metabolism. The increased butyric acid observed in the olive pomace-supplemented group may be attributed to fermentation of the indigestible fiber fraction and phenolic compounds present in olive pomace (54).

This observed increase in propionate in the KE and OP+KE groups and the increase of butyrate in the OP group could be due to the domination of butyrate-producing bacteria (55). Propionate serves as a source of energy and glucose for colonocytes and also functions as an excellent substrate for gluconeogenesis. Butyric acid has been widely recognized for its beneficial effects on intestinal health, including anti-inflammatory properties and promotion of epithelial barrier function (56), potentially contributing to the observed enhancements in intestinal morphology. Volatile fatty acids (VFA) concentrations can range up to 99.8 mmol l-1, depending on the age, physiological condition, and dietary composition of the rabbit. When absorbed, VFAs produced in the cecum can provide approximately 40% of maintenance energy requirements in rabbits. VFAs are the primary source of metabolic energy for the large intestinal mucosa. Consequently, increased VFA synthesis may result in greater energy supply and, thus, improved body weight gain.

The absence of significant caecal pH changes, despite varied VFA profiles among experimental groups, points to effective hindgut buffering. This buffering is vital because ammonia utilization in microbial protein synthesis demands a synchronized supply of energy and NH_3_-N. Conveniently, the ATP produced during VFA creation directly fuels this protein synthesis within microbial cells. Furthermore, according to (40), adding multiple enzymes to rabbit feed may improve the gastrointestinal tract environment, possibly by acidifying cecal contents and stabilizing ammonia nitrogen concentrations.

The histomorphometry analysis revealed significant increases in villus height in both K and OK groups, consistent with findings reported by Yang et al. (46), who documented improved intestinal morphology following enzyme supplementation in broilers. The elongation of intestinal villi represents a beneficial adaptation that expands the absorptive surface area, potentially enhancing nutrient utilization efficiency (41). Intestinal morphology remains an important indicator of proper intestinal absorptive function and activity (57). The height or length of intestinal villi is directly connected to the efficiency of digestion and absorption (58). A study by Cowieson and Bedford (59) reported that exogenous enzyme supplementation increases digestion and nutrient absorption. Additionally, (60) observed an increase in villi height and crypt depth in groups receiving dietary-supplemented exogenous multi-enzymes. These findings align with our observations of increased duodenal villi length in groups treated with Kemzyme alone or with both Kemzyme and olive pomace compared to the control group. The significant enlargement of Brunner's gland area observed in the KE and OP+KE groups suggests enhanced secretory capacity, which may contribute to improved digestion through increased secretion of alkaline mucus that neutralizes gastric acid and protects the duodenal mucosa (61). Interestingly, the group treated with both Kemzyme and olive pomace exhibited larger Brunner's gland areas than the group treated with Kemzyme alone, suggesting a synergistic effect of these dietary interventions on secretory function (62–64). These morphological adaptations collectively support improved intestinal function and may explain the enhanced metabolic parameters observed in our study. The combination of increased villus height and enhanced Brunner's gland secretory capacity would be expected to improve both digestive efficiency and absorptive capacity, potentially explaining the improvements in growth performance and metabolic parameters observed in the supplemented groups.

It is noteworthy that the combined supplementation of olive pomace and Kemzyme (OP+KE group) frequently yielded results comparable to Kemzyme supplementation alone (K group), without apparent synergistic effects in all parameters. This observation suggests that the enzymatic intervention may be the primary driver of the observed physiological improvements, with olive pomace potentially serving as a substrate for enzymatic action rather than an independent modulator (8). However, the economic advantage of partial replacement of yellow corn with olive pomace (a byproduct with lower market value) should not be overlooked, particularly in regions where olive processing generates substantial quantities of pomace (2). From an economic and environmental perspective, the partial replacement of yellow corn with olive pomace, a byproduct of olive oil production, represents a sustainable approach to rabbit nutrition, particularly when combined with exogenous enzyme supplementation. These findings contribute to the growing body of evidence supporting the integration of agricultural byproducts and enzyme technology in sustainable animal production systems.

## 5 Conclusion

This study demonstrates that supplementing New Zealand White rabbits with Kemzyme (0.5%), alone or with olive pomace (10% replacing with corn), improves metabolic parameters, digestive enzyme activities, and intestinal morphology. Specifically, the research observed significant gains in glucose metabolism, protein synthesis, digestive enzyme activities, and intestinal morphology, notably villus height and Brunner's gland area. These positive effects are likely mediated by enhanced nutrient digestibility, gut microbiota modulation, and stimulated intestinal epithelial cell proliferation. The use of olive pomace, an olive oil industry byproduct, offers a sustainable and cost-effective approach to rabbit nutrition, especially when paired with exogenous enzymes to maximize its nutritional potential. The polyphenolic compounds in olive pomace provide antioxidant benefits, while the multi-enzyme complex facilitates the breakdown of complex feed components, leading to better nutrient utilization. Our findings underscore the value of integrating agricultural byproducts and enzyme technology for sustainable animal production systems.

## Data Availability

The raw data supporting the conclusions of this article will be made available by the authors, without undue reservation.
